# Sucrose synthase gene family analysis in loquat reveals *EjSUS4* as a key regulator of sugar accumulation

**DOI:** 10.3389/fpls.2026.1860539

**Published:** 2026-06-09

**Authors:** Junhao Yan, Wenqi Shao, Jie Ding, Minghui Chen, Chaofeng Yang, Kai Xu, Boping Wu

**Affiliations:** 1 College of Horticulture, Zhejiang A&F University, Hangzhou, Zhejiang, China; 2 State Key Laboratory for Development and Utilization of Forest Food Resources, Zhejiang A&F University, Hangzhou, Zhejiang, China

**Keywords:** enzymatic activity, fruit ripening, loquat, sucrose synthase, sugar accumulation

## Abstract

**Introduction:**

Soluble sugar composition critically determines the commercial quality of loquat (*Eriobotrya japonica*), a prominent Rosaceae fruit. Sucrose synthase (SUS) plays a central role in carbohydrate partitioning and fruit sink strength, yet the SUS gene family in loquat remains uncharacterized.

**Methods:**

Seven *EjSUS* genes were identified from the loquat genome and analyzed via phylogenetics, chromosomal mapping, synteny, and structural characterization. Spatiotemporal expression profiling was performed during fruit development in two cultivars (‘YingShuang’ and ‘ZheHong 16’). Soluble sugar contents were measured, and Pearson correlation analysis was conducted. Recombinant *EjSUS4* protein was subjected to in vitro enzymatic assays. Promoter cis-element analysis was also performed.

**Results:**

The seven *EjSUS* genes were classified into three subfamilies. Segmental and tandem duplications drove family expansion. *EjSUS4* (Group I) was specifically and highly upregulated during the terminal ripening stage, coinciding with peak sucrose accumulation, whereas other *EjSUS* members showed very low or undetectable expression. Loquat was confirmed as a hexose-accumulating fruit. *EjSUS4* transcript levels showed a highly significant positive correlation with sucrose content. Recombinant *EjSUS4* exhibited robust sucrose synthesis activity, and its promoter contained ABRE cis-elements potentially linking it to ABA signaling.

**Discussion:**

Our findings demonstrate that *EjSUS4* is the predominant SUS member driving late-stage sugar accumulation in loquat. This work provides valuable genetic resources and a theoretical basis for molecular breeding to improve loquat fruit quality.

## 1 Introduction

Loquat (*Eriobotrya japonica*) is an economically important subtropical evergreen fruit tree of the Rosaceae family, valued for its early ripening season and unique nutritional profile (Jing et al., 2023). Fruit quality, particularly flavor and consumer preference, is largely determined by the accumulation of soluble sugars, with sucrose often acting as the predominant carbohydrate in mature fruits (Ali et al., 2022; Meng et al., 2026). The dynamic balance of sucrose synthesis and degradation not only dictates fruit sweetness but also provides essential carbon skeletons and energy for various physiological processes during development (Liu et al., 2024a). Therefore, elucidating the molecular mechanisms of sucrose metabolism is essential for the genetic improvement of loquat fruit quality.

Sucrose metabolism in plants is governed by a complex enzymatic network, in which sucrose synthase (SUS; EC 2.4.1.13) plays a pivotal role. Unlike invertases that irreversibly hydrolyze sucrose, SUS catalyzes the reversible conversion of sucrose and uridine diphosphate (UDP) into fructose and UDP-glucose (Stein and Granot, 2019). This reaction is widely recognized as a biochemical marker for sink strength (Ruan, 2014). SUS is a key enzyme in plant sucrose metabolism, playing pivotal roles not only in fruit development but also in cellulose synthesis and starch accumulation (Sun et al., 2025). Its activity is essential for maintaining the sucrose balance between source and sink tissues, a fundamental process for carbohydrate partitioning in plants (Julius et al., 2017). The products of SUS-mediated cleavage are directly utilized in starch biosynthesis, cellulose synthesis, and the respiratory pathway (Li et al., 2024). Beyond its metabolic role, emerging evidence suggests that *SUS* and its associated sugar signaling pathways are involved in diverse developmental processes, including floral bud differentiation and responses to abiotic stresses (Yoon et al., 2021; Pan et al., 2025). Recent reviews have further highlighted that SUS functions in concert with alternative pathways to maintain carbon flux in plants, rather than acting as the sole regulator for starch and cellulose synthesis (Dangwal and Suri, 2023).

In the Rosaceae family, the *SUS* gene family has undergone significant functional specialization related to fruit development. In apple (*Malus domestica*), *MdSUS1* and *MdSUS2.1* exhibit high expression in fruit tissues, correlating with rapid sucrose accumulation during ripening (Tong et al., 2018); in peach (*Prunus persica*), *PpSUS1* has been identified as a key factor in regulating sink strength and carbohydrate partitioning (Li et al., 2015); in plum (*P. salicina*), *PsSUS1* plays a critical role in sucrose accumulation across different developmental stages (Han et al., 2026). In pear (*Pyrus bretschneideri*), *PbSUS3* is significantly involved in both stone cell formation and sucrose metabolism (Abdullah et al., 2018); while in strawberry (*Fragaria vesca*), *FvSUS4* has been reported to act as a regulator of fruit ripening and sugar accumulation (Zurn et al., 2020). Furthermore, studies in cherry (*P. avium*) indicate that the coordinated expression of *PaSUS* members maintains the energy supply required for fruit development (Ezura et al., 2017). In loquat, although previous physiological studies have analyzed SUS enzyme activity and gene expression under exogenous treatments (Ali et al., 2022), a systematic identification and evolutionary analysis of the *SUS* family at the whole-genome level remains lacking.

Despite the identified roles of *SUS* genes in other species, several critical questions regarding the loquat *SUS* family remain unresolved, including: How many *SUS* members are present in the loquat genome, and what are their structural and evolutionary relationships compared to those in close relatives like apple and pear? What are the spatiotemporal expression patterns of different *SUS* subfamilies during loquat fruit development? How do these members respond to cultivar-specific or environmental variations in sucrose content? To address these gaps, the present study utilized the recently published high-quality chromosome-level genome assembly of wild loquat (Jing et al., 2023) to perform the first comprehensive identification of the *EjSUS* gene family. By integrating bioinformatics, transcriptomics, and multi-omics association analyses, we characterized the physicochemical properties, collinearity, and expression profiles of *EjSUS* genes. These results not only enrich our understanding of the molecular mechanisms of sucrose metabolism in Rosaceae but also provide an important theoretical basis and candidate gene resources for the regulation of sugar content in loquat breeding.

## 2 Materials and methods

### 2.1 Plant materials

In this study, two loquat cultivars differing in ripening behavior were selected: ‘YingShuang’, a white-fleshed early-maturing variety (harvested around May 10th), and ‘ZheHong 16’, a red-fleshed mid-season variety (harvested around May 25th). Fruits were collected at four developmental stages according to (Liu et al., 2024b; Zhang et al., 2025a).color For each stage, three biological replicates (five fruits per replicate) were immediately snap-frozen in liquid nitrogen and stored at -80 °C for subsequent analyses.

### 2.2 Identification of *EjSUS* gene family members in loquat

The genome data of loquat were retrieved from the GigaScience Database (http://gigadb.org/dataset/view/id/100711). Genome sequences of apple (*M. domestica*), peach (*P. persica*), grape (*Vitis vinifera*), and tomato (*Solanum lycopersicum*) were downloaded from the NCBI database (https://www.ncbi.nlm.nih.gov/). Amino acid sequences of *SUS* family members from *Arabidopsis thaliana* were obtained from The Arabidopsis Information Resource (TAIR, https://www.arabidopsis.org/). To identify *SUS* members in the loquat genome, we downloaded the hidden Markov model (HMM) profile of the conserved sucrose synthase domain (PLN00142) from the PFAM database (http://pfam.sanger.ac.uk/). A BLAST-based search was then performed to screen for candidate loquat *SUS* genes. Sequences containing the PLN00142 domain were retained using the NCBI Conserved Domain Database (CDD, http://www.ncbi.nlm.nih.gov). The physicochemical properties of the deduced SUS proteins, including molecular weight, isoelectric point, and amino acid length, were calculated using the ExPASy server (https://web.expasy.org/protparam/). The subcellular localization of EjSUS proteins were predicted via the WoLFPSORT platform (https://wolfpsort.hgc.jp/).

### 2.3 Phylogenetic analysis across multiple species

Using MEGA 11.0 software, multiple sequence alignments were carried out on SUS protein sequences derived from loquat, apple (*M. domestica*), *A. thaliana*, tomato (*S. lycopersicum*), peach (*P. persica*), and grape (*V. vinifera*). A phylogenetic tree was reconstructed via the neighbor-joining (NJ) method, with branch support evaluated by 1,000 bootstrap replicates. The resulting tree was then visualized and annotated using the Interactive Tree of Life (iTOL) online platform (https://itol.embl.de/).

### 2.4 Gene structure, conserved motifs and chromosomal localization

The exon–intron structures and chromosomal localization of loquat *SUS* family genes were visualized and analyzed using TBtools software based on the GFF annotation file. Conserved motifs in the corresponding proteins were identified with the online tool MEME (https://meme-suite.org/). Chromosomal localization of the identified loquat *SUS* members was determined using TBtools software, based on the positional information extracted from the loquat genome annotation file.

### 2.5 Collinear analysis

The One-Step MCScanX-SuperFast algorithm within TBtools was used to investigate intragenomic collinearity among loquat *SUS* genes, while the Advanced Circos module of the same software was employed to visualize intergenomic syntenic relationships across species.

### 2.6 Promoter cis-element analysis

A 2000 bp region immediately upstream of the start codon was extracted with TBtools and subsequently subjected to cis−regulatory element prediction through PlantCARE (https://bioinformatics.psb.ugent.be/webtools/plantcare/html/). The built−in graphics tools of TBtools were then used to visualize all detected regulatory elements.

### 2.7 RNA extraction and transcriptome sequencing

Total RNA was extracted from loquat fruits using the CTAB method, and samples with RIN ≥ 7.0, OD260/280 between 1.8 and 2.0, and RNA quantity ≥ 1 μg were used for library preparation. Poly(A)^+^ mRNA was enriched with oligo(dT) beads, fragmented, and reverse transcribed into double−stranded cDNA using M−MuLV reverse transcriptase and DNA polymerase I. After end−repair, A−tailing, and adapter ligation, the libraries were quantified with a Qubit, assessed on a Bioanalyzer, and subjected to 150 bp paired−end sequencing on a NovaSeq 6000 platform. Raw reads were trimmed with fastp (v0.20.0), mapped to the reference genome using HISAT2 (v2.0.5), and transcript levels were calculated as FPKM with StringTie (v1.3.3b). Differential expression analysis was carried out with DESeq2 (v1.20.0) using an adjusted p−value < 0.05 and |log2FoldChange| > 1.

### 2.8 Measurement of sugar content

For sugar quantification, 0.1 g fruit samples were homogenized in 1.4 mL methanol and incubated at 70 °C for 15 min. After centrifugation at 11,000 × g for 10 min, 1.5 mL distilled water and 750 μL chloroform were added to the supernatant to induce phase separation, followed by another centrifugation at 2,200 × g for 10min. The aqueous layer (1 mL) was collected and stored at −80 °C. For derivatization, 100 μL of the extract was mixed with 10 μL of ribitol solution (0.2 mg/mL) as internal standard, dried under nitrogen, and then derivatized in two steps. First, 60 μL of methoxyamine hydrochloride (20 mg/mL in pyridine) was added and the mixture was shaken at 950 rpm for 1.5 h at 37 °C. Subsequently, 40 μL of BSTFA containing 1% TMCS was added and reaction continued for another 30 min under the same conditions. The derivatized samples were transferred to GC vials and analyzed by gas chromatography within 24 h to ensure sample stability.

### 2.9 Protein structure prediction, subcellular localization, and enzyme activity assay

The three−dimensional structure of EjSUS4 was predicted by homology modeling using SWISS−MODEL.

For subcellular localization, the coding sequence of *EjSUS4* was cloned into the Super1300 vector, the *EjSUS4*−GFP fusion construct was transiently expressed in *Nicotiana benthamiana* leaves via *Agrobacterium* infiltration, and GFP fluorescence was observed under a confocal microscope.

The sucrose synthesis activity of purified recombinant EjSUS4 protein was measured using a commercial SS−II Assay Kit (Comin Biotechnology Co., Ltd., Suzhou, China), following the manufacturer’s instructions. Briefly, the purified protein was incubated with the reaction buffer and substrates at 25 °C for 10 min. The colorimetric reagent was then added, and the mixture was boiled for 10 min. Subsequently, the chromogenic solution was added, followed by boiling for another 30 min. The absorbance of the final reaction mixture was measured at 480 nm. One unit of activity was defined as 1 µg sucrose produced per minute per milligram of protein.

### 2.10 Statistical analysis

Graphical representations were generated using OriginPro 2023 and ChiPlot (https://www.chiplot.online/). Pearson correlation analysis was performed to evaluate the linear relationships between transcript levels of *SUS* genes and the contents of sucrose, glucose, and fructose. A threshold of p < 0.05 was used to determine statistical significance. In the resulting heatmaps, significant correlations are distinctly marked, with correlation coefficients (R-values) indicating both the magnitude and direction of the associations.

## 3 Results

### 3.1 Genome-wide identification and physicochemical properties of *EjSUS* genes

A comprehensive genome-wide screening identified a total of seven *SUS* genes in the loquat (*E. japonica*) genome, which were named *EjSUS1* to *EjSUS7* based on their chromosomal locations. The physicochemical properties of the seven EjSUS proteins were analyzed (**Table 1**). The amino acid lengths ranged from 702 (EjSUS5) to 913 (EjSUS3), with molecular weights varying from 79.58 kDa (EjSUS5) to 103.53 kDa (EjSUS3). The theoretical isoelectric points (pI) ranged from 5.95 (EjSUS4) to 6.79 (EjSUS6), with a mean pI of 6.34, indicating that all EjSUS proteins are slightly acidic. The instability index varied from 29.78 (EjSUS4) to 43.54 (EjSUS3), with most members classified as stable (instability index < 40), except for EjSUS3 which was slightly unstable. The aliphatic index ranged from 85.84 (EjSUS1) to 96.78 (EjSUS5), suggesting good thermal stability. The grand average of hydropathicity (GRAVY) values were all negative, ranging from -0.339 (EjSUS2) to -0.111 (EjSUS5), indicating that all EjSUS proteins are hydrophilic. Notably, EjSUS4 exhibited the lowest instability index (29.78) and a relatively low pI (5.95), suggesting it may be a particularly stable and acidic member of the family. Subcellular localization prediction for EjSUS4 assigned the highest score to the plasma membrane, although a cytoplasmic localization was also possible. For the remaining members, EjSUS2, EjSUS6, and EjSUS7 were predicted to be localized in the cytosol, whereas EjSUS1, EjSUS3, and EjSUS5 were predicted to be localized in the chloroplast.

**Table 1:** Physicochemical property analysis of SUS family proteins.

EjSUS proteins	Number of amino acids (aa)	Molecular weight (kDa)	Theoretical pI	Instability index	Aliphatic index	Grand average of hydropathicity	Subcellular localization prediction
EjSUS1	899	101.28	6.46	38.21	85.84	-0.264	chloroplast
EjSUS2	818	92.71	6.64	31.7	87.98	-0.339	cytosol
EjSUS3	913	103.53	6.38	43.54	89.81	-0.161	chloroplast
EjSUS4	807	92.29	5.95	29.78	94.21	-0.266	plasma membrane
EjSUS5	702	79.58	6.02	35.48	96.78	-0.111	chloroplast
EjSUS6	893	101.27	6.79	38.24	86.82	-0.29	cytosol
EjSUS7	785	88.89	6.16	35.36	86.97	-0.337	cytosol

### 3.2 Phylogenetic analysis and chromosomal localization of *EjSUS* genes

**Figure 1:**
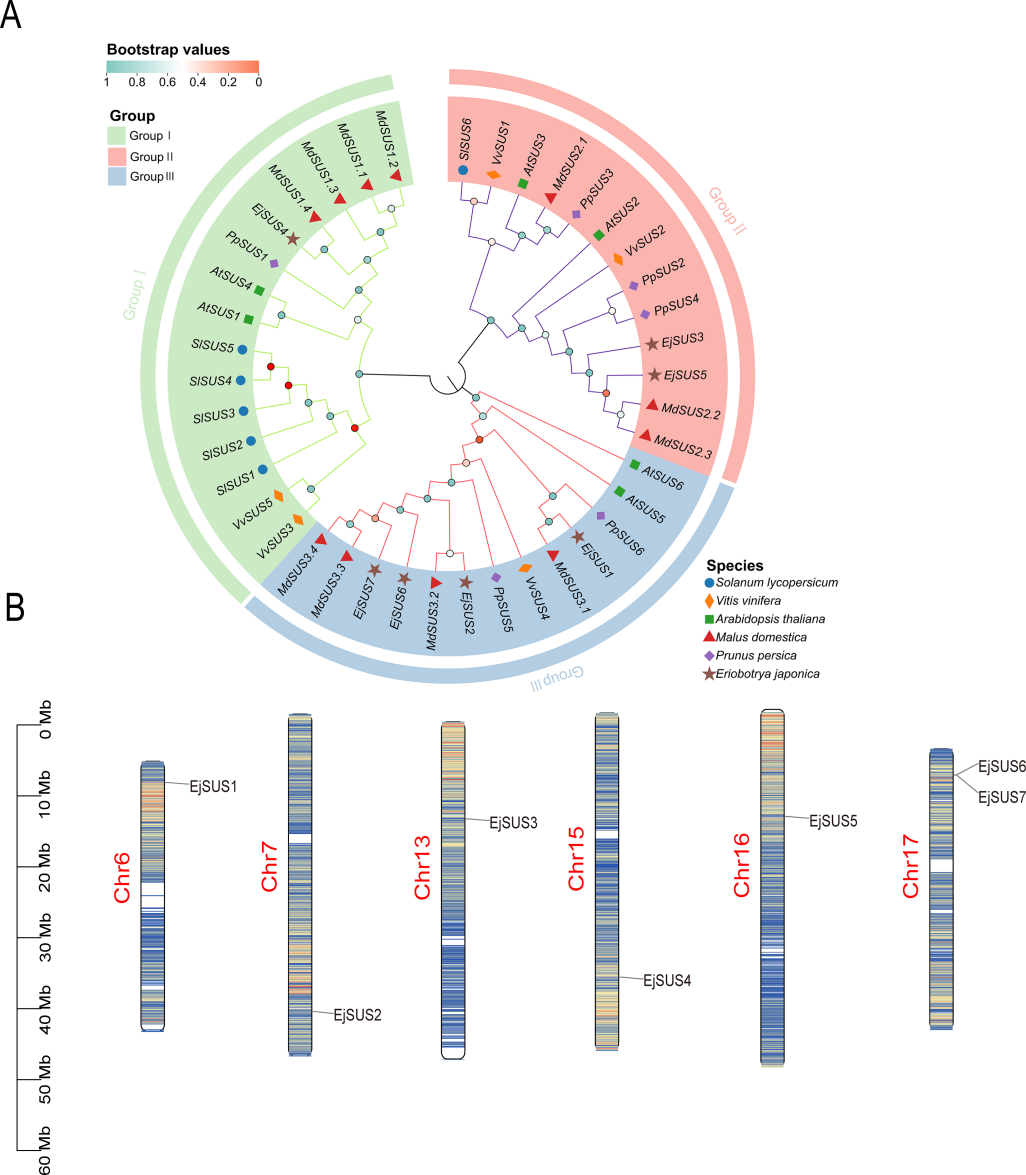
Phylogenetic relationships and chromosomal distribution of *SUS* genes in loquat. **(A)** Phylogenetic analysis of the *SUS* family across multiple species. **(B)** Chromosomal localization of *SUS* genes in loquat.

To investigate the evolutionary relationships of the *SUS* gene family in loquat, we constructed a phylogenetic tree using the deduced amino acid sequences of the seven EjSUS proteins and their orthologs from *A. thaliana*, *V. vinifera*, *M. domestica*, and *P. persica* (**Figure 1A**). The tree clearly partitioned all *SUS* members into three distinct subfamilies (SUS I, SUS II, and SUS III), consistent with the established classification in angiosperms.

The SUS I clade (Group I) was located at the top of the tree and contained *EjSUS4*, which clustered closely with apple *MdSUS1.1*~*1.4*, peach *PpSUS1*, and Arabidopsis *AtSUS1/4*. Notably, *EjSUS4* showed the closest evolutionary relationship with *MdSUS1.4* (bootstrap value = 100), suggesting a conserved orthologous relationship within the Maleae tribe. The SUS II clade (Group II) occupied the middle section and included *EjSUS3* and *EjSUS5*, which were orthologous to *AtSUS2* and the *MdSUS2* group. The SUS III clade (Group III) was positioned at the bottom and contained the remaining four loquat members: *EjSUS1*, *EjSUS2*, *EjSUS6*, and *EjSUS7*. These clustered with *AtSUS5/6* and *MdSUS3* homologs. The majority of nodes, especially those involving *EjSUS4*, had bootstrap values of 90–100, indicating high reliability of the tree topology.

The seven *EjSUS* genes were unevenly distributed across six chromosomes: *EjSUS4* on Chr15, *EjSUS3* on Chr13, *EjSUS5* on Chr16, *EjSUS1* on Chr6, *EjSUS2* on Chr7, and *EjSUS6/7* on Chr17 (**Figure 1B**).

### 3.3 Conserved motif and gene structure analysis of *EjSUS* genes

**Figure 2:**
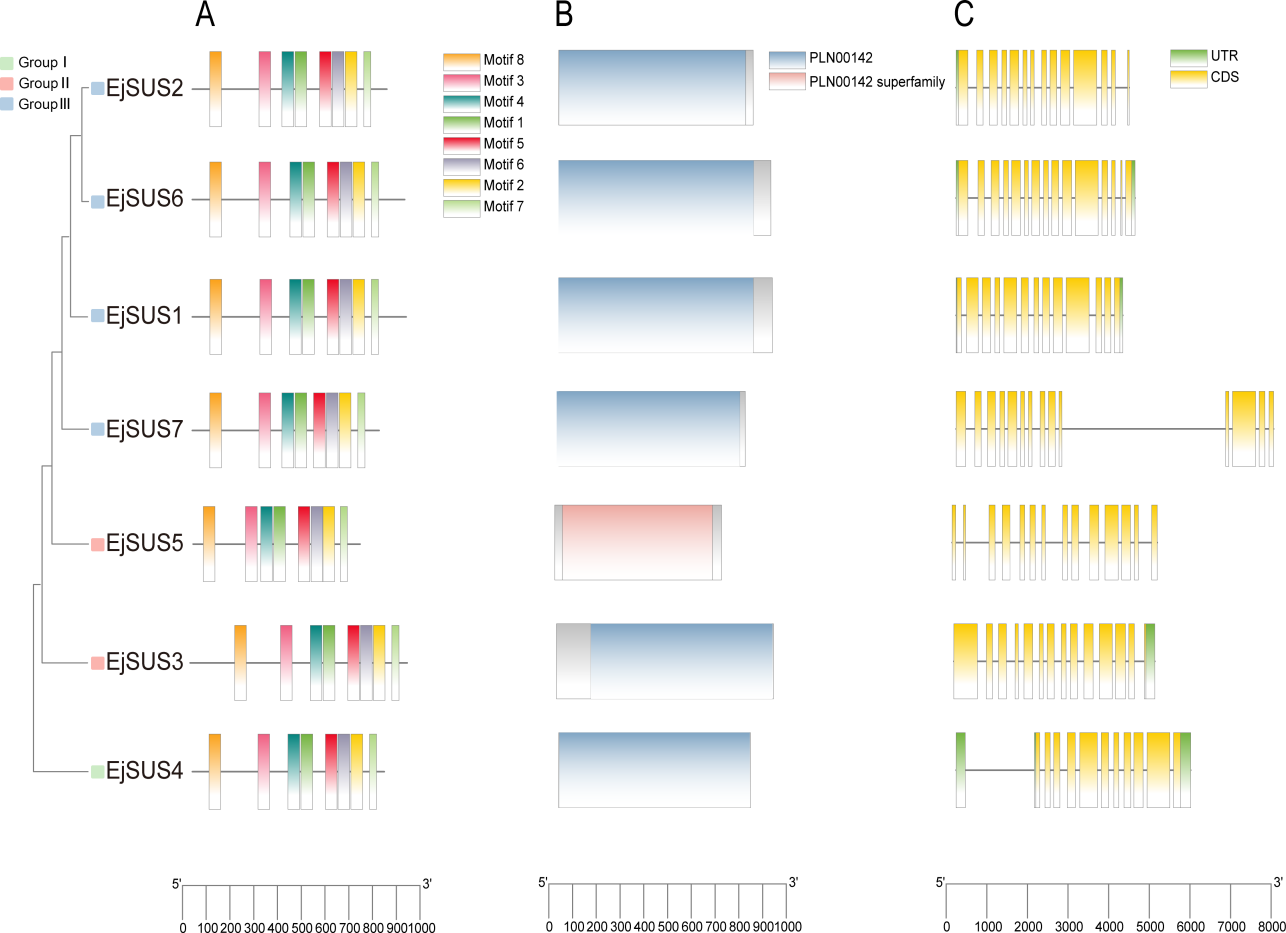
Motifs, domains, and gene structures of the *SUS* genes in loquat. **(A)** Distribution of conserved motifs in loquat SUS proteins. **(B)** Protein domain architectures of loquat *SUS* genes. **(C)** Exon-intron structures of loquat *SUS* genes.

We analyzed the conserved motifs and exon–intron organization of the seven *EjSUS* genes to characterize structural divergence among the three subfamilies (**Figure 2**). All EjSUS proteins contained the core catalytic motifs (motifs 1–7), confirming their identity as true sucrose synthases (PLN00142). However, the distribution of motif 8 and the gene length varied significantly across subfamilies.

In Group I (*EjSUS4*), we observed a complete and highly ordered motif arrangement, representing the canonical architecture for the SUS I clade. The gene structure of *EjSUS4* consisted of 13–15 exons with relatively large introns, particularly the first and second introns, which was typical for Rosaceae SUS I genes. In Group II (*EjSUS3* and *EjSUS5*), we detected the highest degree of structural divergence. Both genes exhibited more compact intron–exon arrangements compared to Group I. In Group III (*EjSUS1*, *EjSUS2*, *EjSUS6*, and *EjSUS7*), the motif patterns were remarkably uniform and almost identical to the Group I standard, suggesting strong conservation of catalytic potential. However, gene structure showed dramatic variation: *EjSUS7* contained an exceptionally large central intron spanning over 5,000 bp, while *EjSUS6* and *EjSUS2* displayed nearly identical structures, supporting the hypothesis of a recent gene duplication event.

### 3.4 Analysis of cis-acting elements in the promoter of *EjSUS* genes

**Figure 3:**
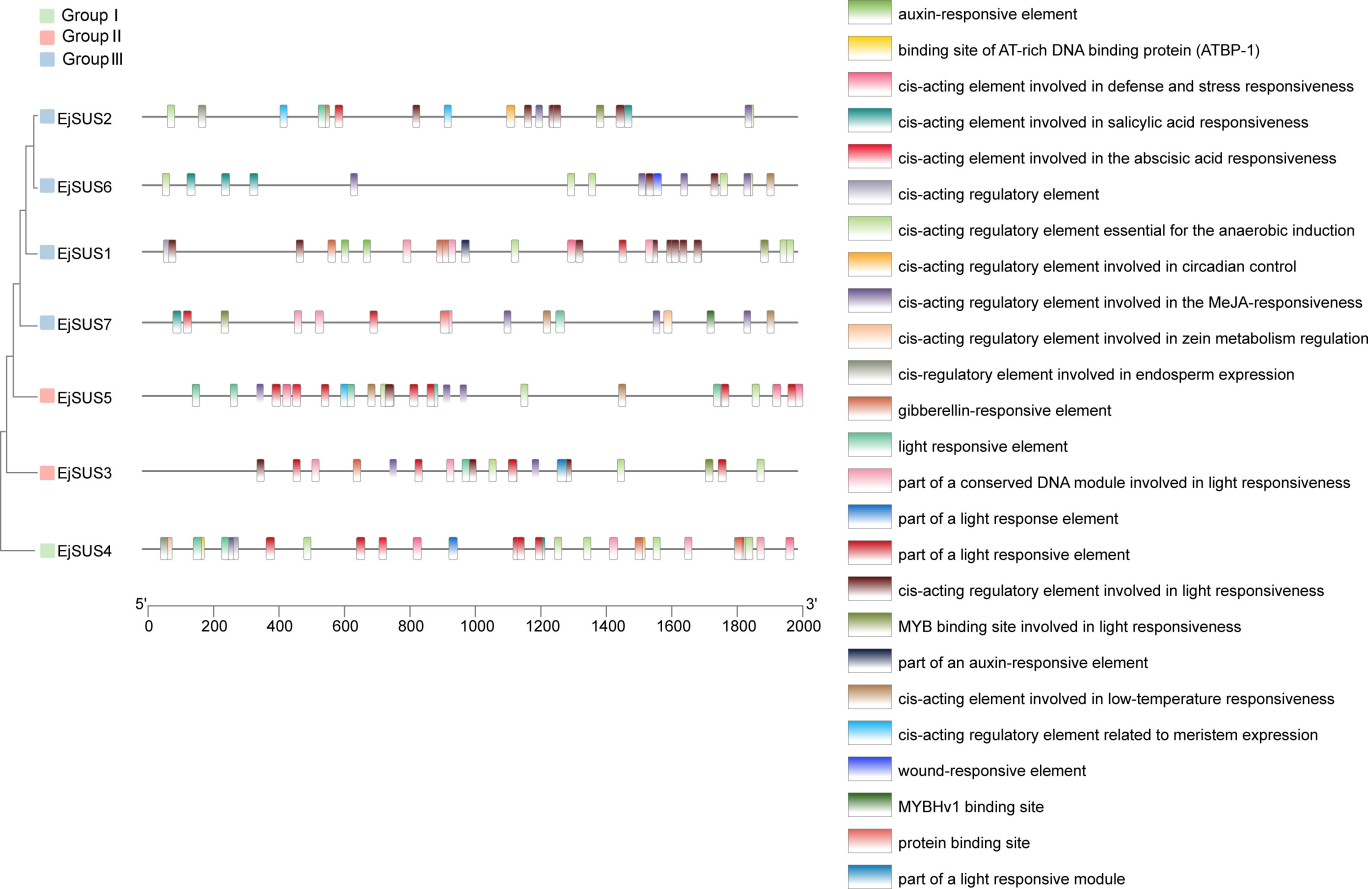
Cis-acting elements in the promoter regions of *SUS* genes in loquat. Boxes of different colors represent distinct types of cis-acting elements.

To gain insight into the potential regulatory mechanisms of *EjSUS* genes, we analyzed the cis-acting elements in the 2,000 bp promoter regions upstream of the start codon (**Figure 3**). The results revealed distinct enrichment patterns that correlated with the phylogenetic subfamilies.

In Group I (*EjSUS4*), the promoter was highly enriched with abscisic acid (ABA)-responsive elements (ABREs), light-responsive modules (G-box, Box 4), and auxin-responsive elements. In Group II (*EjSUS3* and *EjSUS5*), the promoters showed a strong presence of methyl jasmonate (MeJA)-responsive elements (in *EjSUS3*), salicylic acid (SA)-responsive elements (in *EjSUS5*), and TC-rich repeats associated with defense and stress responsiveness. In Group III, we observed the highest diversity of regulatory elements. *EjSUS2* contained elements involved in circadian control; *EjSUS7* harbored low-temperature responsive elements (LTRs); and *EjSUS6* was notably rich in anaerobic induction elements (ARE). These findings suggested that Group I members were primarily involved in ripening-associated sugar accumulation, while Group II and III members may participate in stress signaling and adaptive responses.

### 3.5 Synteny analysis of the *EjSUS* gene family

**Figure 4:**
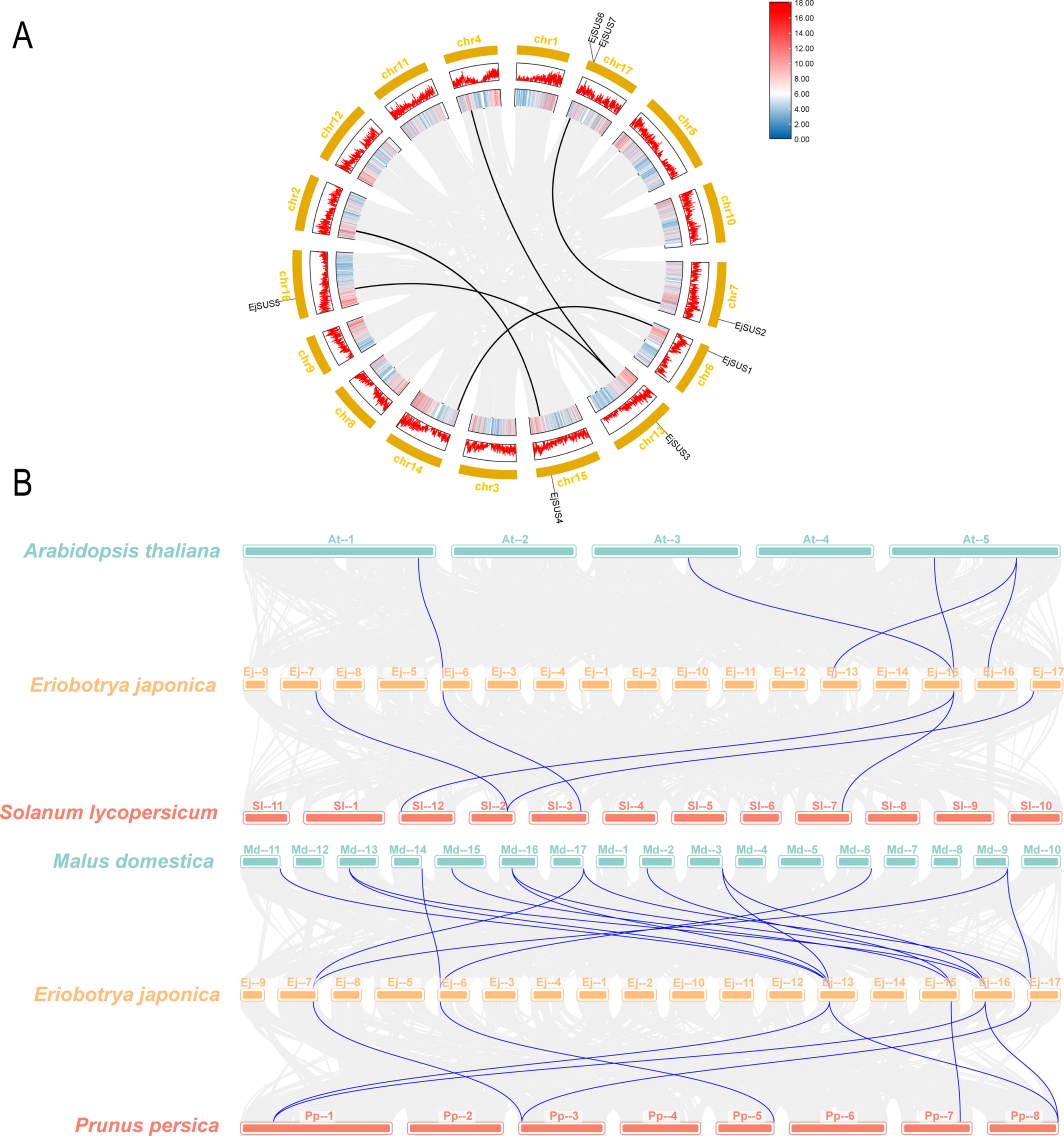
Collinearity analysis of the *SUS* gene family in loquat. **(A)** Intraspecific collinearity analysis of *SUS* genes in loquat. Blue lines represent collinear relationships among *SUS* genes. **(B)** Comparative collinearity analysis of *SUS* genes between loquat and five representative species. Blue lines depict collinear gene pairs.

To elucidate the expansion mechanisms of the *EjSUS* gene family, we performed intraspecific collinearity analysis (**Figure 4A**). The results revealed multiple segmental duplication events among different chromosomal regions. A significant syntenic link was observed between Chr7 (*EjSUS2*) and Chr17 (*EjSUS6/7*), indicating that *EjSUS2* and the genomic segment containing the tandemly duplicated *EjSUS6/7* originated from a segmental duplication event. Notably, *EjSUS3* (Chr13) exhibited complex collinearity: it showed a clear syntenic relationship with *EjSUS5* (Chr16), and also linked to a region on Chr4, suggesting remnants of ancient whole-genome duplication or polyploidization events. Additionally, *EjSUS1* (Chr6) was connected to a syntenic block on Chr14, although no *SUS* gene was annotated at the corresponding locus on Chr14. Strikingly, *EjSUS4* (Chr15) showed no syntenic links to any other *EjSUS* member. This observation suggests that *EjSUS4* was an evolutionarily distinct and relatively isolated member, which likely did not arise from recent segmental or tandem duplications in the loquat genome.

Interspecific synteny analysis compared loquat with *A. thaliana*, *S. lycopersicum*, *M. domestica*, and *P. persica* (**Figure 4B**). Synteny was significantly higher within the Rosaceae family than between loquat and more distantly related species. Group I (*EjSUS4*) exhibited the most stable orthologous relationships across all five species, with direct syntenic links to apple Md-Chr13, peach Pp-Chr7, and Arabidopsis At-Chr5. Group II showed more selective synteny: *EjSUS3* (Chr13) was orthologous to Md-Chr13 and At-Chr5, while *EjSUS5* (Chr16) showed a distinct link to Pp-Chr8. Group III provided evidence of the Maleae-specific WGD, with nearly identical links between loquat Chr17 (*EjSUS6/7*) and apple Chr9, and between loquat Chr7 (*EjSUS2*) and apple Chr17. The 1-to-2 relationship between loquat/apple chromosomes and peach chromosomes was a classic signature of the polyploidization event in the ancestor of loquat and apple.

### 3.6 Transcriptome profiling of *EjSUS* genes

**Figure 5:**
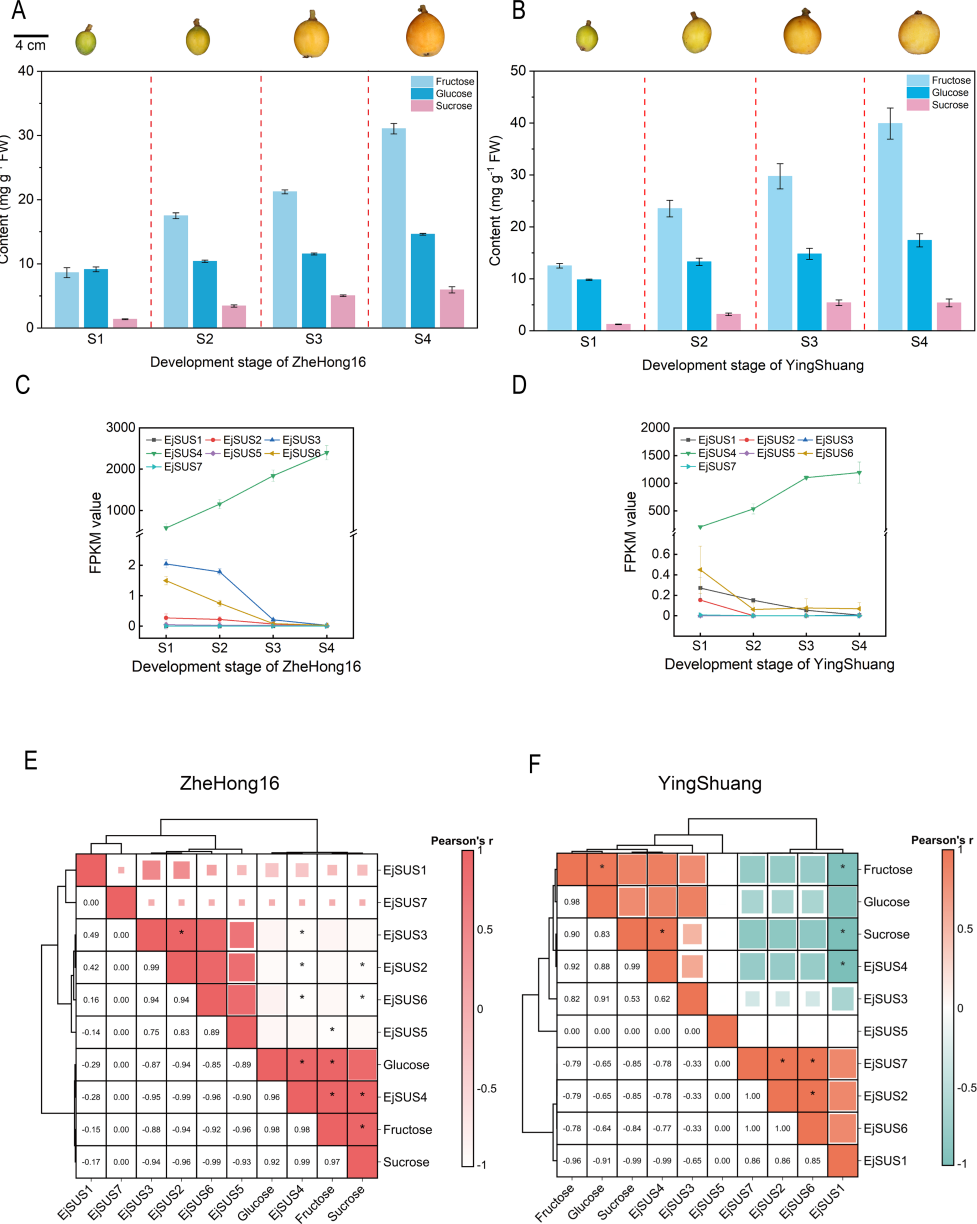
Soluble sugar content and *EjSUS* genes expression in two loquat cultivars across fruit developmental stages, with correlation analysis between gene expression and sugar accumulation. **(A, B)** Fructose, glucose, and sucrose contents in ‘ZheHong 16’ **(A)** and ‘YingShuang’ **(B)**. **(C, D)** RNA-seq-based expression of *EjSUSs* in ‘ZheHong 16’ **(C)** and ‘YingShuang’ **(D)**. **(E, F)** Pearson correlation coefficients between *EjSUSs* expression and sucrose, glucose, or fructose content in ‘ZheHong 16’ **(E)** and ‘YingShuang’ **(F)**.

**Figure 6:**
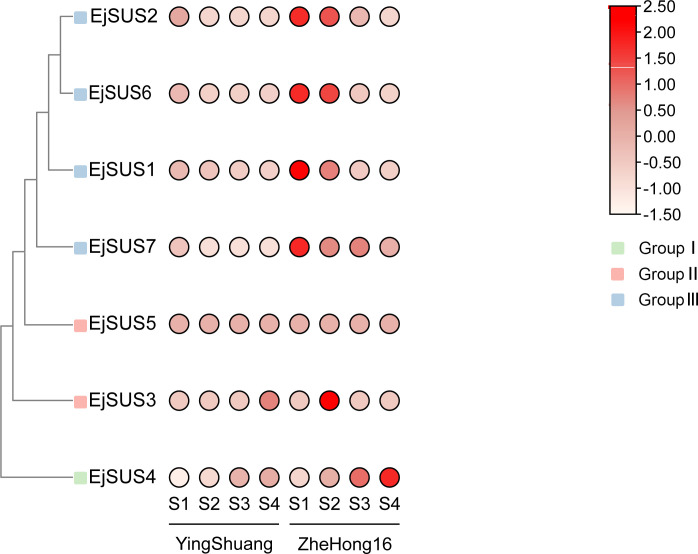
Transcriptome profiling of two loquat cultivars across fruit developmental stages. among.

The expression patterns of *EjSUS* genes were examined by RNA−seq data (**Figures 5C, D**). The expression profiles clearly segregated according to the three phylogenetic subfamilies (**Figure 6**). Group I (*EjSUS4*) exhibited a classic ripening-associated expression pattern. In both cultivars, transcript levels were relatively low during early stages (S1–S2) and reached their maximum at S4, the final ripening stage. Notably, the peak at S4 was significantly higher in ‘ZheHong 16’ than in ‘YingShuang’, which correlated with the higher sucrose accumulation in ‘ZheHong 16’. This sharp upregulation of *EjSUS4* at S4 coincided with the late-phase increase in sucrose and the peak of hexose accumulation, strongly suggesting that *EjSUS4* is a key driver of late-stage sugar synthesis.

Group II (*EjSUS3* and *EjSUS5*) and Group III (*EjSUS1*, *EjSUS2*, *EjSUS6*, and *EjSUS7*) showed very low or near−undetectable expression levels throughout fruit development in both cultivars. *EjSUS5* was almost silent across all stages, while *EjSUS3* exhibited a marginally higher but still extremely low level, with a very slight transient elevation at S1 in ‘ZheHong 16’. Similarly, all Group III members displayed minimal transcript abundance at all four stages, without any clear early−stage dominance. These results indicated that among the seven *EjSUS* members, only *EjSUS4* was consistently expressed at appreciable levels in fruit tissues and was therefore the predominant candidate for regulating sucrose accumulation during loquat fruit ripening.

### 3.7 Correlation between sugar accumulation and *EjSUS* gene expression

To identify candidate *SUS* genes involved in fruit sugar accumulation, we analyzed the soluble sugar content across four developmental stages (S1 to S4) in two loquat cultivars, ‘YingShuang’ and ‘ZheHong 16’ (**Figures 5A, B**). In both cultivars, fructose was the most abundant soluble sugar at all stages, followed by glucose, while sucrose remained at relatively low concentrations throughout development. This pattern indicates that loquat was predominantly a hexose-accumulating fruit. In ‘YingShuang’, fructose levels increased continuously from S1 to S4, reaching approximately 40 mg g^-1^ FW at S4. In ‘ZheHong 16’, fructose also accumulated steadily but reached a slightly lower peak (~30 mg g^-1^ FW). Sucrose levels remained low during early stages (S1–S2) and exhibited a modest increase from S3 to S4 in both cultivars.

To further elucidate the relationship between *EjSUS* transcriptional activity and sucrose metabolism, we performed Pearson correlation analysis between the expression levels of each *EjSUS* member and the contents of sucrose, glucose, and fructose across four developmental stages in both ‘YingShuang’ and ‘ZheHong 16’ cultivars (**Figures 5E, F**).

A highly significant positive correlation was observed between *EjSUS4* and sucrose accumulation in both cultivars, with correlation coefficients of r = 0.99 (p < 0.01) in ‘YingShuang’ and r = 0.99 (p < 0.01) in ‘ZheHong 16’. In contrast, Group III members exhibited strong negative correlations with sucrose content. In ‘YingShuang’, *EjSUS1* showed a strong negative correlation (r = -0.99, p < 0.01) and *EjSUS6* showed a moderate negative correlation (r = -0.84, p < 0.05); in ‘ZheHong 16’, *EjSUS6* (r = -0.99, p < 0.01) and *EjSUS2* (r = -0.96, p < 0.01) also displayed significant negative correlations. Notably, Group III genes (*EjSUS2*, *EjSUS6*, and *EjSUS7*) displayed extremely high positive correlations with one another within each cultivar, with coefficients often exceeding r = 0.85 or reaching r = 1.00, indicating robust co-expression among these tandemly and segmentally duplicated members. Additionally, *EjSUS4* expression was strongly correlated with glucose and fructose, further supporting its key role in sugar accumulation.

### 3.8 Structural modeling, enzymatic activity, and subcellular localization of EjSUS4

**Figure 7:**
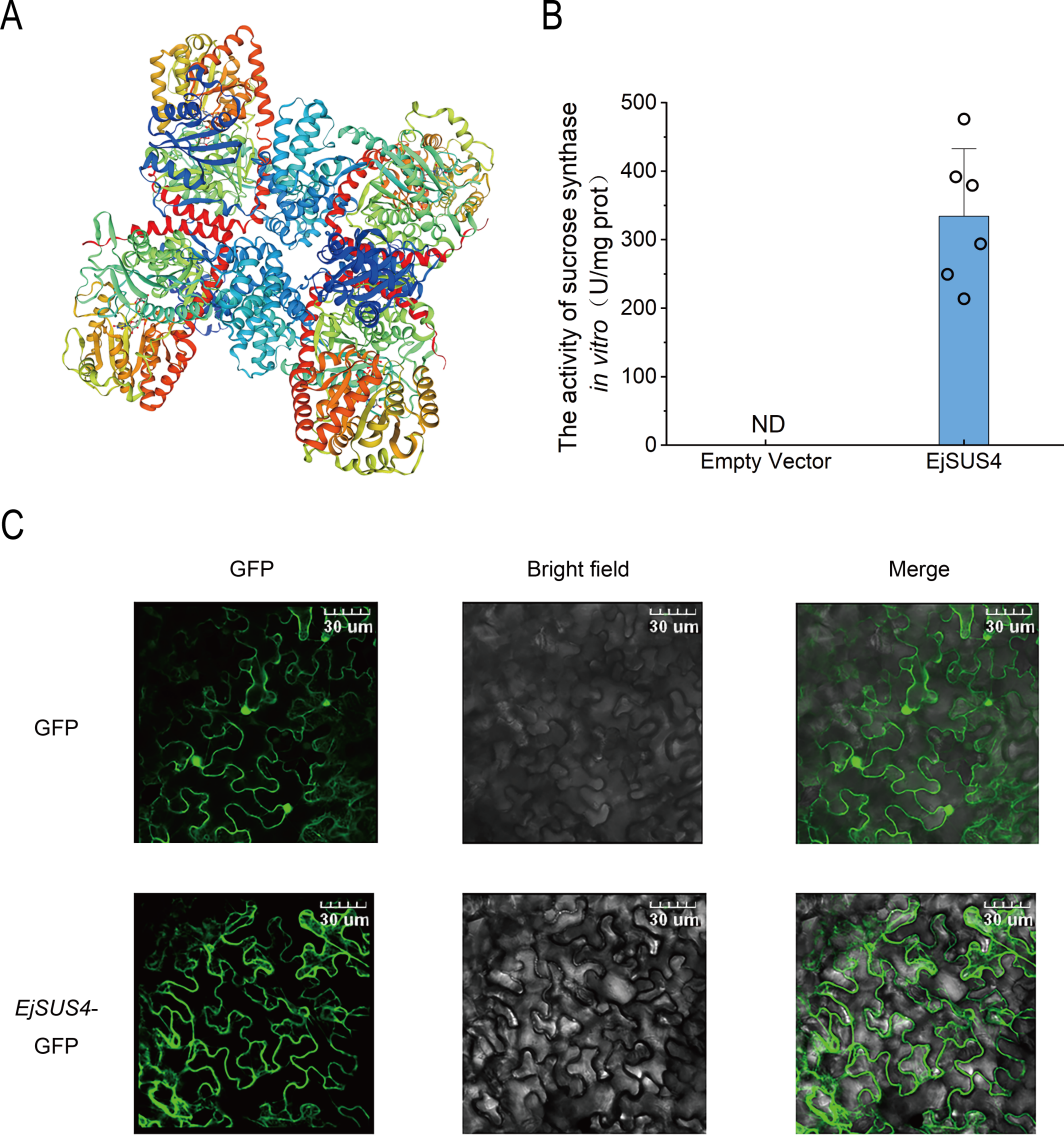
Structural modeling, *in vitro* enzymatic activity, and subcellular localization of EjSUS4 in *Nicotiana benthamiana* leaves. **(A)** Predicted three-dimensional structure of EjSUS4 tetramer. **(B)** Sucrose synthesis activity of recombinant EjSUS4 measured *in vitro*, with empty vector control. **(C)** Subcellular localization of EjSUS4 in *Nicotiana benthamiana* leaves.

To gain structural insight into the function of EjSUS4, we performed protein homology modeling and analyzed the predicted three-dimensional structure (**Figure 7A**). The model revealed that EjSUS4 adopted a tetrameric quaternary structure, consisting of four identical subunits assembled into a stable symmetrical core. This tetrameric formation was essential for enzymatic stability and biological function, consistent with the known behavior of sucrose synthases in plants.

To validate the biochemical function of EjSUS4, we purified the recombinant protein and assessed its *in vitro* enzymatic activity in the sucrose synthesis direction (**Figure 7B**). The assay demonstrated that EjSUS4 possessed robust synthetic activity, producing sucrose at an average rate of approximately 330 U/mg prot. The empty vector control showed no detectable activity, confirming that the sucrose produced in the experimental group was entirely dependent on the intact EjSUS4 tetramer. These results confirmed that EjSUS4 was a fully functional enzyme exhibiting robust *in vitro* sucrose synthesis activity.

To investigate the subcellular distribution of EjSUS4, we transiently expressed an EjSUS4-GFP fusion protein in *Nicotiana benthamiana* leaves. The empty vector was used as a control. Confocal microscopy analysis revealed that the control GFP fluorescence was observed in both the nucleus and the plasma membrane (**Figure 7C**). In contrast, the EjSUS4-GFP fusion protein exhibited a distinct localization pattern, with fluorescence detected in both the plasma membrane and the cytoplasm. No fluorescence was observed in the nucleus. The merged images of bright-field and dark-field channels confirmed the membrane-localized signal of EjSUS4-GFP. These results demonstrated that EjSUS4 is associated with both the plasma membrane and the cytoplasm in plant cells.

## 4 Discussion

The identification of seven *SUS* genes in the loquat genome provides a foundational framework for understanding sucrose metabolism in this Maleae species. This number is slightly lower than that reported in apple (11 *MdSUS* genes) but comparable to the *SUS* family in peach (6 *PpSUS* genes). Phylogenetic analysis categorized the *EjSUS* members into three distinct subfamilies (SUS I, II, and III), a classification that remains highly conserved across angiosperms (Stein and Granot, 2019; Xu et al., 2019). Notably, the clustering of *EjSUS4* within the SUS I group highlights a conserved evolutionary trajectory within the Maleae tribe, likely linked to the ancient whole-genome duplication (WGD) event shared by loquat and apple (Jing et al., 2023). Synteny analysis revealed that segmental duplication, rather than tandem duplication, was the primary driver of *EjSUS* family expansion. This expansion mechanism allows for potential subfunctionalization, although the functional fate of duplicated copies may vary across species (Li et al., 2024).

Functional specialization in gene families is often reflected in structural divergence. While core catalytic domains were strictly maintained across all *EjSUS* members, we observed subfamily-specific variations in motif distribution and intron patterns. For instance, the exceptionally large central intron in *EjSUS7* (Group III) suggests distinct structural evolution within these clades. Similar structural variations in other horticultural crops have been associated with altered catalytic efficiency or tissue-specific targeting (Duan et al., 2021). Beyond soluble sugar metabolism, SUS is often associated with the cell wall and plasma membrane to provide UDP-glucose for cellulose and callose synthesis, as observed in tobacco pollen tubes (Persia et al., 2008). This supports the structural divergence we observed in the *EjSUS* family, which may relate to localized carbon partitioning. However, expression analysis revealed that in fruit tissues, only *EjSUS4* (Group I) was consistently expressed at appreciable levels, while other *EjSUS* members showed very low or undetectable expression across all developmental stages. This suggests that, within the context of fruit sugar metabolism, *EjSUS4* is the predominant functional member, whereas other *EjSUS* paralogs may play minor roles or function in non-fruit tissues or under specific environmental conditions.

The subcellular localization analysis suggested that EjSUS4 is associated with both the plasma membrane and the cytoplasm. This observation is consistent with recent findings in other horticultural crops. In tea plant (*Camellia sinensis*), CsSUS1 and CsSUS3 were shown to localize to both the plasma membrane and the cytosol (Huang et al., 2025). Similarly, in lily (*Lilium*), LoSuSy and LoSuSy4 were predominantly localized to the plasma membrane, with LoSuSy4 also exhibiting a faint cytoplasmic signal (Pan et al., 2025). These collective findings align well with our observation that EjSUS4 is distributed in both the plasma membrane and the cytoplasm. Given its localization and physicochemical properties, EjSUS4 is associated with the regulation of sucrose synthesis. Future co-localization experiments using a standard plasma membrane marker would help to unequivocally confirm this dual localization.

Soluble sugar analysis revealed that loquat is a hexose-accumulating fruit, with fructose and glucose dominating throughout development. Nevertheless, a modest but consistent increase in sucrose content was observed during the final stage (S4), coinciding with the sharp upregulation of *EjSUS4* in both cultivars. This temporal correlation, together with the verified *in vitro* sucrose synthesis activity of EjSUS4, supports the role of Group I *SUS* members as key regulators of ripening-associated sink strength, as documented in apple and pear (Tong et al., 2018; Meng et al., 2026). Notably, ‘ZheHong 16’ exhibited higher *EjSUS4* transcript levels at S4 compared to ‘YingShuang’, which correlated with its higher sucrose accumulation, suggesting that transcriptional variation of this gene contributes to cultivar-specific sugar profiles. Similar to our findings in loquat, stage-specific gene expression driving carbohydrate changes has been documented in Chinese cherry (Shang et al., 2022). Furthermore, recent studies in strawberry have shown that transcriptional regulators, such as NAC and MADS-box proteins, antagonistically coordinate sucrose accumulation (Xiao et al., 2025), suggesting a complex regulatory network for SUS-mediated sugar flux in Rosaceae fruits.

Promoter cis-element analysis revealed that *EjSUS* genes are regulated by diverse phytohormone and stress signals. The enrichment of ABRE elements in the *EjSUS4* promoter is particularly noteworthy, as abscisic acid (ABA) is a master regulator of ripening in non-climacteric fruits like loquat (Ali et al., 2022). This promoter architecture likely explains the ripening-specific induction of *EjSUS4*, suggesting that an ABA-dependent pathway mediates the late-stage sucrose increase. This is consistent with recent findings in cotton, where ABA was shown to promote assimilate transport by directly inducing the expression of SUS and acid invertase genes (Zhang et al., 2025b). This is consistent with reports that sugar signaling and ABA pathways interact to regulate metabolic flux in other species (Yoon et al., 2021; Pan et al., 2025). Although Group II and III promoters contained stress-related elements (MeJA, SA, LTR, ARE), their very low expression in fruit tissues implies that these genes may be primarily involved in stress responses or metabolic processes in other organs rather than in fruit sugar accumulation. This inference is supported by multiple lines of evidence: SUS3 has been shown to enhance cold tolerance in tomato (Li et al., 2024), and ZmSUS1 overexpression improves drought resistance in maize by elevating soluble sugar content (Xiao et al., 2024). The identification of SUS families in other crops like melon under nutrient stress (Shah et al., 2025) and the role of carbohydrate partitioning in rice drought response (Xu et al., 2015) further suggest that the non-ripening-related EjSUS genes in loquat may function primarily in environmental adaptation.

In summary, this study provides the first genome-wide identification of the *SUS* gene family in loquat. Our results demonstrate that the *EjSUS* family expanded primarily through segmental duplication, followed by structural divergence. However, among the seven members, only *EjSUS4* (Group I) is highly expressed during fruit ripening and exhibits robust *in vitro* sucrose synthesis activity, strongly suggesting that it acts as the primary driver of late-stage sugar accumulation in loquat fruit. These findings enrich our understanding of sucrose metabolism in Rosaceae and provide a candidate target (*EjSUS4*) for molecular breeding to improve loquat fruit quality.

## 5 Conclusion

In this study, we performed the first genome-wide identification and characterization of the sucrose synthase (*SUS*) gene family in loquat (*E. japonica*). A total of seven *EjSUS* genes were identified and classified into three subfamilies (SUS I, II, and III) based on phylogenetic analysis. Synteny analysis revealed that segmental duplication, together with tandem duplication of *EjSUS6/7* on Chr17, drove the expansion of this gene family. Structural analysis showed that all EjSUS proteins retained the core catalytic motifs, while subfamily-specific variations (e.g., the large intron in *EjSUS7*) suggested divergent evolutionary trajectories.

Expression profiling across four fruit developmental stages in two cultivars demonstrated that only *EjSUS4* (Group I) was consistently expressed at appreciable levels, with a sharp peak at the final ripening stage (S4). The other six *EjSUS* members showed very low or undetectable expression in fruit tissues, implying that they may function in non-fruit organs or under specific stress conditions. Pearson correlation analysis revealed a strong positive correlation between *EjSUS4* expression and sucrose accumulation (r = 0.99, p < 0.01), and *in vitro* enzymatic assays confirmed that *EjSUS4* possesses robust sucrose synthesis activity (approximately 330 U/mg prot). Promoter cis-element analysis further supported the ripening-specific regulation of *EjSUS4* through ABA-responsive elements.

Collectively, our results indicate that *EjSUS4* is the predominant *SUS* member driving late-stage sucrose accumulation in loquat fruit, while other *EjSUS* paralogs play minor or context-specific roles. This study not only enriches our understanding of *SUS* family evolution in Rosaceae but also provides a candidate genetic target (*EjSUS4*) for molecular breeding to improve fruit sweetness and quality in loquat.

## Data Availability

All data generated or analyzed in this study are included in this published article and supplementary information files. The datasets analyzed during the current study are available in the NCBI Sequence Read Archive database (project number: PRJNA1109454). Further inquiries can be directed to the corresponding author.
